# Rehabilitation report of 2 cases of spinal cord ischemic injury after intra-aortic repair

**DOI:** 10.1097/MD.0000000000038852

**Published:** 2024-07-26

**Authors:** Shu Cheng, Xuan Li, Yufei Zhang, Chenxi Liu, Yi Rao, Yang Zhang, Jinlun Wu, Jinwen Sun, E-Liisa Laakso

**Affiliations:** a Department of Rehabilitation Medicine, Wuhan University of Science and Technology Affiliated Wuhan Resources & Wisco General Hospital, Wuhan, China; b Medical School, Wuhan University of Science and Technology, Wuhan, China; c Department of Pain Medicine, Wuhan University of Science and Technology Affiliated Wuhan Resources & Wisco General Hospital, Wuhan, China; d Mater Research, South Brisbane, QLD, Australia.

**Keywords:** cerebrospinal fluid drainage, intra-aortic repair, radicular artery, rehabilitation, spinal cord ischemic injury

## Abstract

**Rationale:**

Spinal cord ischemia injury is a serious complication after intra-aortic surgery, with a low incidence but high disability rate. However, patients often do not receive comprehensive treatment in the early stages of the disease. Therefore, active neurological intervention is needed to protect and prevent spinal cord ischemia during and after surgery. In this paper, rehabilitation program and imaging data of 2 cases with spinal cord ischemic injury are presented and discussed regarding causes, prevention and acute treatment with this disease, which could be referred by clinicians.

**Patient concerns:**

Case report 1: A 69-year-old male patient underwent aortic arch aneurysm and thoracic endovascular aortic repair (coated stent) was performed under general anesthesia. Complete paralysis of both lower limbs, constipation, and urinary retention occurred after surgery and was subsequently referred to our rehabilitation department. Case report 2: A man aged 41 years experienced sudden chest pain with no dizziness or headache. Weakness of both lower limbs gradually appeared over 30 minutes with subsequent loss of consciousness. He was diagnosed with aortic dissection and underwent aortic stent implantation. Inpatient rehabilitation began systematically 3 months after discharge.

**Diagnoses:**

The 2 patients were diagnosed with paraplegia and spinal cord ischemic injury.

**Interventions:**

The patients received strength and transfer training, sensory input, health mission, and activities of daily living.

**Outcomes:**

Patient 1 returned home without assistive devices and patient 2 returned home with wheelchair.

**Lessons:**

Perioperative spinal cord protection is directly related to postoperative quality of life. Once the symptoms of spinal cord ischemic injury occur, cerebrospinal fluid drainage should be performed as soon as possible to increase mean arterial pressure. At the same time, methylprednisolone, ganglioside, anticoagulation, vasodilator drugs, and symptomatic supportive treatments are required. Intercostal artery and subclavian artery are reconstructed if necessary. Symptom stability flags referral to commence rehabilitation. Repetitive functional training is necessary to help patients return to the family and society as soon as possible.

## 1. Introduction

Endovascular surgery is the main treatment method for aortic dissections, aortic aneurysms, and other aortic diseases.^[1]^ Spinal cord ischemic injury is one of the most serious complications of endoluminal treatment of the aorta, and studies have reported 2.5% to 10% incidence of spinal cord ischemic injury caused by thoracic endovascular aortic repair.^[2,3]^ Neurological function after mild spinal cord ischemia injury can be recoverable after rehabilitation treatment, while severe ischemic injury could lead to paraplegia. At present, there are few reports on the rehabilitation treatment of spinal cord ischemic injury complicated by thoracic endovascular aortic repair. Our department admitted 2 patients with spinal cord ischemic injuries because of intra-aortic repair from June 2023 to December 2023. Informed consent was obtained from both patients prior to presentation of the clinical and imaging details. One patient was paraplegic after stenting for aortic aneurysm,^[4]^ and the other was paraplegic after stenting for aortic dissection (Standford B).^[5]^ Both patients were evaluated and treated with systematic rehabilitation. After 3 months of rehabilitation treatment, the muscle strength of both lower limbs of case 1 recovered well, and urinary and bowel function was normal. Minimal sensation recovery was identified in the lower limbs of case 2, and there was no active recovery of urinary and bowel incontinence. In this paper, rehabilitation program and imaging data of 2 cases with spinal cord ischemic injury are presented and discussed regarding causes, prevention, and acute treatment with this disease, which could be referred by clinicians.

## 2. Case reports

### 2.1. Treatment section

Case report 1: A 69-year-old retired male patient underwent computerized tomography angiography of thoracic aorta and showed aortic arch aneurysm. After completing relevant examination, thoracic endovascular aortic repair (coated stent) was performed under general anesthesia. Complete paralysis of both lower limbs, constipation, and urinary retention occurred after surgery. Left subclavian stent implantation was performed after 24 hours, and postoperative indwelling catheterization, ganglioside, and circulatory support were given. The muscle strength of both lower limbs partially recovered, and the patient could sit and stand independently for a short time but could not walk independently when assessed for discharged 2 weeks after surgery. After discharge, the man was transferred to comprehensive rehabilitation.

**Figure 1.**
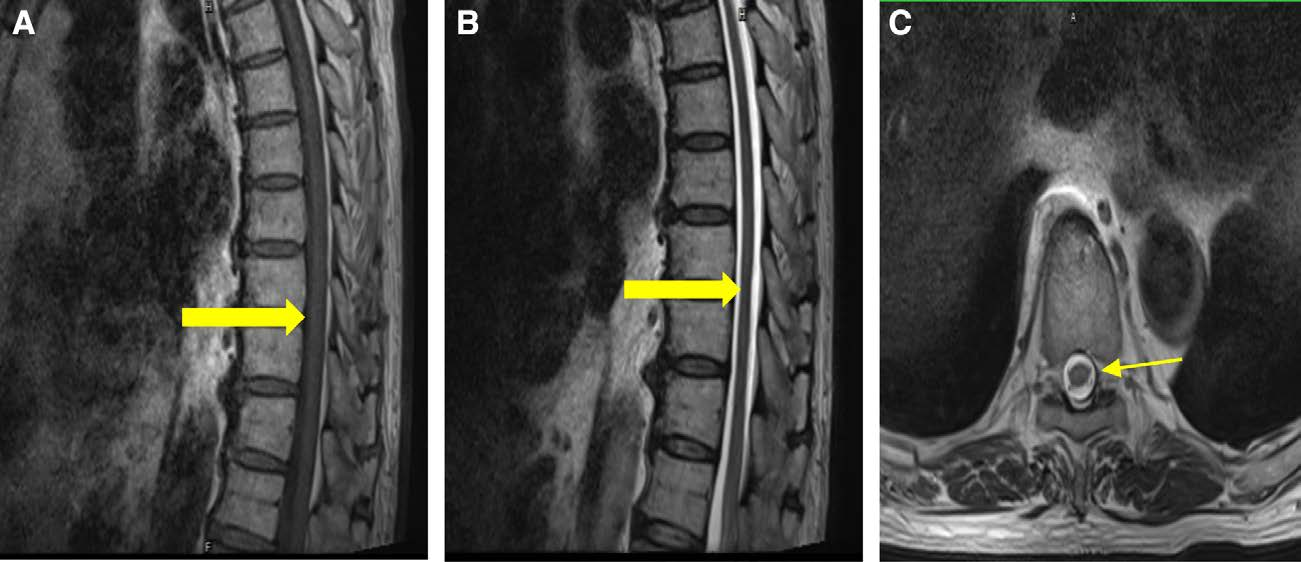
Case 1 (A) sagittal T2WI show no lesion in the thoracic medulla. (B) Sagittal T1WI show no lesion in the thoracic medulla. (C) Axial T2WI l show no lesion in the thoracic medulla at the level of T8/9 intervertebral level.

On admission to rehabilitation, specialist physical examination identified a positive bulbar cavernous reflex, sensation bilaterally to T11, motor responsiveness at L2, and American Spinal Injury Association (ASIA) degree C.^[6]^ A thin magnetic resonance imaging (MRI) scan of the thoracic pulp showed no obvious lesions in the thoracic medulla (Fig. 1). Drug therapies were prescribed to control blood pressure (levamlodipine, oral tablet 2.5 mg qd), as antiplatelet aggregation (aspirin tablet 100 mg qd, clopidogrel tablet 75 mg qd), for lipid-lowering (atorvastatin calcium tablet 20 mg qn), for neurotrophic effects (mecobalamin tablet 0.5 mg tid), stabilize heart rate (metoprolol sustained-release Tablet 47.5 mg qd). The overall goals of rehabilitation were to strengthen the lower limbs and core muscles, sensory training, sit-standing transfer and walking training, and bladder and rectal sphincter retraining. The patient’s goals were independent walking and normal defecation.

With family involvement, standing training occurred twice a day, for 30 minutes each time with the goal of improving the muscle strength of the abdomen, back, and lower extremities.^[7]^ After 8 weeks, when the muscle strength was above grade 3, progressive resistance training with an elastic band was prescribed, especially for key muscles of the lower limbs, lower back, and gluteus muscles.^[8]^ Balance and transfer training included standing balance training, center of gravity transfer training, and bilateral hip, knee, ankle joint control training. Concomitantly, abdominal breathing training was undertaken. At 10 weeks, aerobic treadmill training commenced with the aim of increasing cardiopulmonary endurance. After 2 weeks of commencement of treadmill training, the patient had increased running time from 8 minutes to 20 minutes. Proprioceptive input was included in a walking program utilizing surfaces of different hardness and providing varying sensory stimulus inputs.^[9]^ In response to the problem of urinary retention, the patient kept a voiding diary and was taught how to perform intermittent self-catheterization.^[10]^ As the patient’s urinary control returned, the interval of catheterization was gradually extended to reduce the risk of infection and trauma. By changing food intake and dietary structure (including a regimen of vegetables, fruits, protein, and water intake), constipation improved.

Case report 2: A man aged 41 years experienced sudden chest pain with no dizziness or headache. Weakness of both lower limbs gradually appeared over 30 minutes with subsequent loss of consciousness. He presented to the emergency department for thoracic aortic computerized tomography angiography which showed aortic dissection. The patient was transferred to the ICU of a superior hospital on account of secondary liver insufficiency, renal failure, pulmonary infection, and pulmonary edema. After stabilization, the patient underwent aortic stent implantation under local anesthesia. Postoperatively, the patient had no sensation or active movement in either lower extremity, and he presented with bowel and urine dysfunction. As there was no immediate improvement in his condition, the patient was discharged to home to await rehabilitation during which time he continued intermittent outpatient rehabilitation.

**Figure 2.**
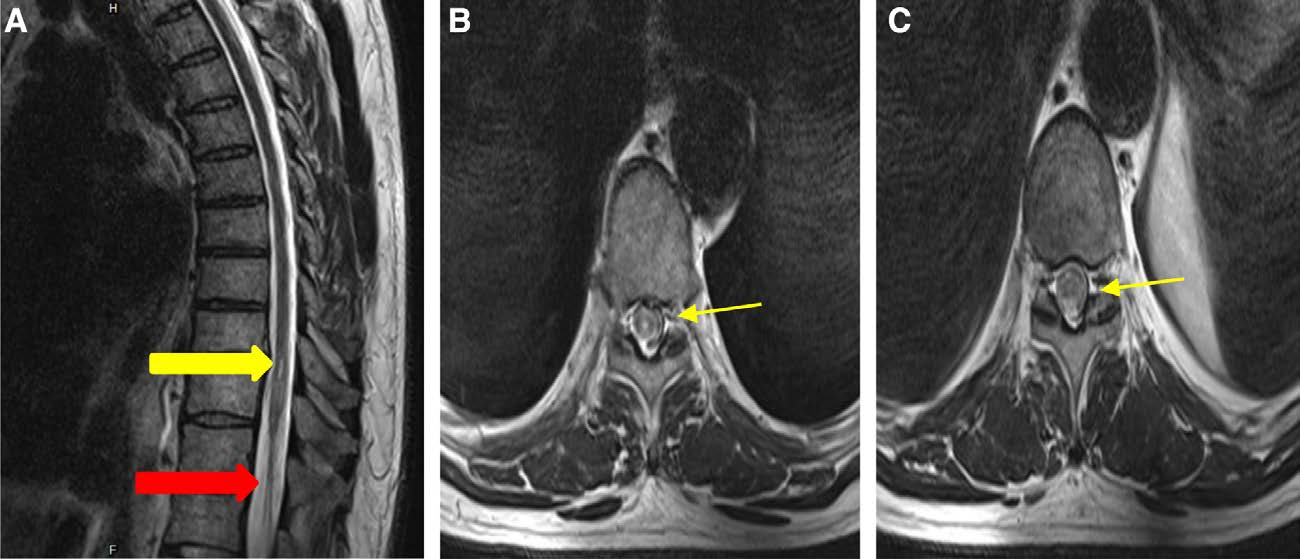
Case 2 (A) sagittal T2WI yellow arrow show T9/10 intervertebral horizontal syringomyelia; red arrows indicate abnormal spinal cord signal changes at T11–12 vertebral level. (B) Yellow arrows on axial T2WI indicate syringomyelia at T9/10. (C) Yellow arrows on axial T2WI indicate abnormal spinal cord signal changes at T11–12 vertebral level.

Inpatient rehabilitation began systematically 3 months after discharge. On admission to rehabilitation, specialist physical examination found that the sensorimotor function of both upper limbs was normal, the bulbar-cavernous reflex was not responsive, the muscle strength of both lower limbs was 0, and joint contracture of both hips and ankles was observed. There was complete absence of sensation in both legs, and incontinence of stool and urine. A pressure sore of 5*3 cm was noted on the left buttock. The bilateral sensory plane was T6 and he was assessed as ASIA A. Due to long-term sitting, the visual analogue scale pain at the waist was 6/10 points. A thin MRI scan of the thoracic medulla showed T9/10 intervertebral horizontal syringomyelia, T10–12 vertebral level spinal atrophy with ischemic changes (Fig. 2). The patient was prescribed oral antiplatelet aggregation (clopidogrel tablet 75 mg qd), lipid-lowering (atorvastatin Tablet 20 mg qn), neurotrophics (mecobalamin tablet 0.5 mg tid), inducing bradycardia (metoprolol extended-release Tablet 47.5 mg qd). Pressure sore dressing changes and comprehensive rehabilitation treatment was prescribed.

The goals of rehabilitation were to prevent complications, increase upper limb muscle endurance, guide the use of rehabilitation assistive devices, improve cardiopulmonary function, and bladder and rectal function re-training. Guided good limb placement instructions were commenced while the patient was in bed to promote sensory input and facilitate pressure sore healing.

Once a day on weekdays, each joint of the lower limbs was moved about 20 times in each axial direction to relieve joint contracture. Special attention was paid to hip adduction and Achilles tendon extension 2 sets each time, each set of 30 seconds, and family members were instructed to repeat the movements once every 4 hours. The muscle strength training was focused on upper limb endurance and core trunk stability. Free weights (dumbbell) were used for strength training in all directions of the upper limbs, 15 sets/muscle group, 2 groups each time. Abdominal curls, lateral bending torso, prone posture and upright training, 10 sets/muscle group, 2 groups each time, twice a day. Stand with instrumented partial weightbearing once a day for 20 minutes each time. Functional activity training (rolling over, bed to chair transfer, wheelchair downhill training) occurred once a day, for 10 minutes each time. Neuromuscular electrical stimulation of the legs was instituted to reduce muscle atrophy.^[11]^ Pneumatic therapy prevented venous thrombosis and reduced swelling of lower limbs.^[12]^ The occupational therapist guided the patient on activities of daily living, including donning and doffing of clothes, shoes and socks with assistance, and stool and urine management.

### 2.2. Outcome section

**Figure 3.**
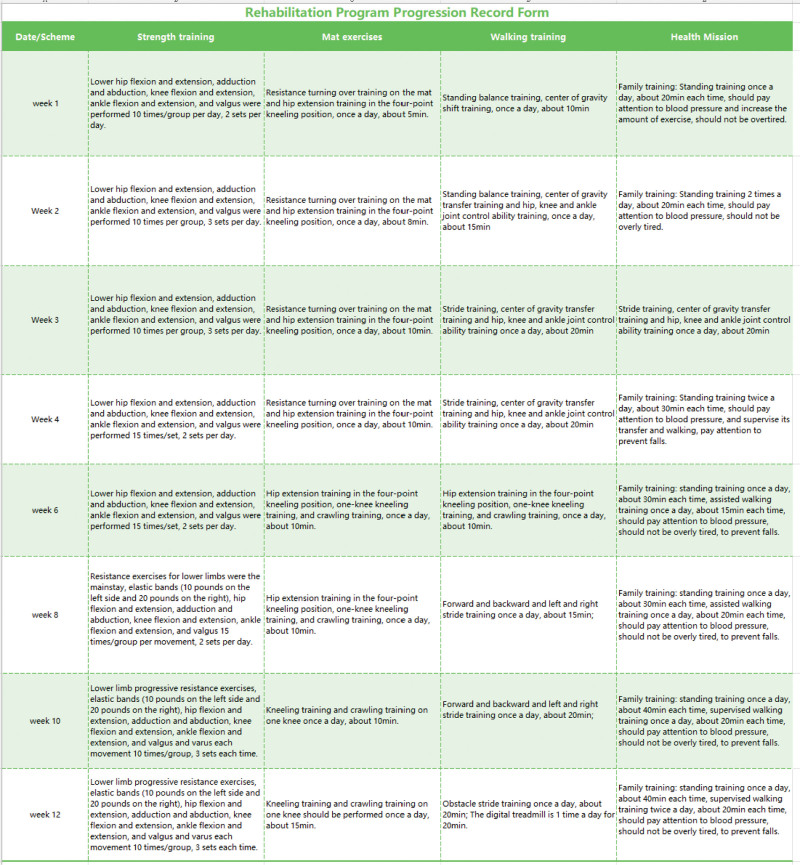
Rehabilitation program progression record form for case 1.

Case report 1: The progress record of the rehabilitation program is shown in Figure 3. After 3 months of rehabilitation, the muscle strength of both lower limbs of the patient was significantly improved (the iliopsoas muscle and quadriceps muscle from grade 3‐ to grade 4, tibialis anterior muscle from grade 3‐ to grade 3+) and the bilateral sensory level changed from T11 to L3, ASIA from C to D, and Barthel Index improved from 55 to 85 points.

**Figure 4.**
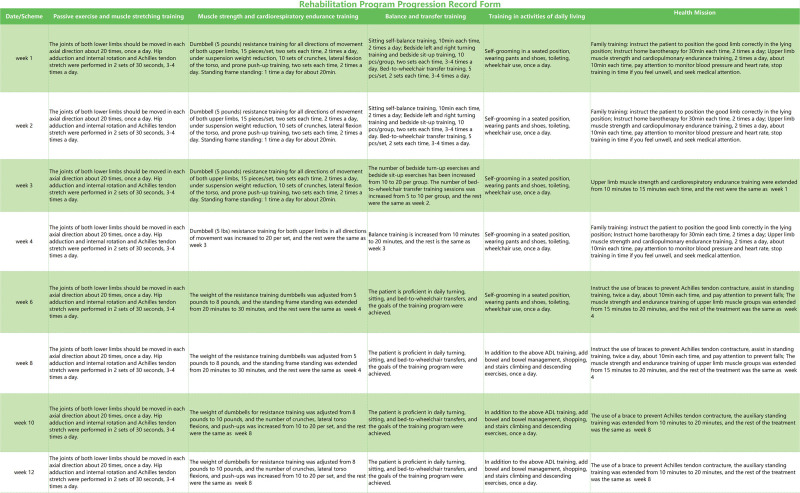
Rehabilitation program progression record form for case 2.

Case report 2: The progress record of the rehabilitation program is shown in Figure 4. After 3 months of rehabilitation, the patient’s upper limb and core muscle endurance and cardiopulmonary endurance were enhanced. Sitting time increased from 30 minutes to 2 hours. He could independently operate the wheelchair, undertake pressure relief strategies, and complete transfers independently from bed–chair. The bilateral sensory plane changed from T6 to T8 and ASIA grade from A to B. The patient’s back pain during sitting was significantly relieved, from visual analogue scale 6/10 to 2/10 points and Barthel Index score from 30 to 50 points, along with healing of the pressure ulcer.

## 3. Discussion

Spinal cord ischemic injury after intracavitary aortic repair is not a common presentation in rehabilitation settings. Patients with spinal cord ischemic injury are prone to bilateral limb sensory and motor dysfunction, bladder and sphincter dysfunction. Therefore, the rehabilitation process of patients with spinal cord ischemic injury after endovascular repair requires comprehensive interprofessional management by the vascular, neurology, rehabilitation, urology, and cardiology teams. Uni-disciplinary knowledge, expertise and treatment experience of this disease can be limited.

The blood supply of the spinal cord is complicated, and the main blood vessels are the anterior and posterior spinal arteries which emanate from the vertebral arteries. The anterior spinal artery branches mainly supply the anterior 2/3 of the spinal cord and are related to spinal motor conduction. The originating branch of the posterior spinal artery mainly supplies the posterior 1/3 of the spinal cord and is related to the deep sensory conduction of the spinal cord—ischemia causes deep paresthesia. During the descending course of the anterior and posterior spinal arteries, many anterior and posterior radicular arteries are joined, the largest of which is the artery of Adamkiewicz originating from the aorta. The artery of Adamkiewicz is commonly located between the T8-L4 vertebrae levels on the left side. The anastomotic branches of the thoracic spinal artery are less than those of the cervico-lumbar artery.^[13,14]^ Therefore, thoracic spinal cord ischemia causes more severe symptoms than ischemia at other segments, especially when the length of the covered stent extends to T8 or below, paraplegia is more likely to occur. Some scholars have divided the causes of ischemia-related spinal cord injury into 3 categories: (1) the degree and duration of spinal cord ischemia, (2) inability to restore spinal cord blood supply, (3) spinal cord ischemia–reperfusion injury.^[15]^ In addition, hypotension, previous spinal surgery, and preoperative acute anemia may aggravate spinal cord ischemia and hypoxia, leading to paraplegia after spinal cord ischemic injury.^[16,17]^

Early detection and treatment are the key points of spinal cord ischemic injury. Cerebrospinal fluid drainage was the most effective method for the prevention and treatment of paraplegia after endovascular aortic repair.^[18]^ The spinal perfusion pressure was equal to the difference between mean arterial pressure and cerebrospinal fluid pressure.^[19–21]^ Early cerebrospinal fluid drainage reduces the cerebrospinal fluid pressure and increases the spinal perfusion pressure to relieve symptoms. However, some scholars have pointed out that preoperative preventive cerebrospinal fluid drainage might increase the risk of craniocerebral infection and low cranial pressure.^[22]^ An important method to alleviate ischemia–reperfusion injury was mitigation of oxidative stress. Methylprednisolone inhibits the process of lipid oxidation and reduces the release of inflammatory factors, thus playing a neuroprotective role.^[23]^ Gangliosides inhibit stress-mediated neuronal apoptosis by protecting cell membranes.^[24]^ Supportive treatments such as anticoagulation and vasodilator drugs were also important in the acute phase of spinal cord ischemic injury.^[25]^ Reconstruction of the intercostal artery and subclavicular artery, neuroelectrophysiological monitoring,^[26]^ ischemic preconditioning,^[27]^ and appropriate improvement of intraoperative and postoperative mean arterial pressure effectively reduce postoperative spinal cord ischemia.^[28^,^29]^

The rehabilitation principles and prognosis of spinal cord ischemic injury after intracavitary aortic repair are like those of spinal cord injury caused by spinal injury, tumor, and myelitis. Different causes of spinal cord injury should be attended to during rehabilitation treatment. In this study, neither of the 2 patients underwent cerebrospinal fluid drainage after spinal cord ischemic injury.

In case 1, incomplete spinal cord injury occurred after intracavitary aortic repair, without obvious lesion of spinal cord in the MRI examination. Through early comprehensive rehabilitation, the muscle strength of both lower limbs was significantly improved, and the patient was able to return to independent self-care. We believe that rehabilitation therapy had a significant effect on the recovery of motor function of the limbs when there was incomplete spinal cord injury after endovascular aortic repair. In case 2, the spinal cord injury occurred in the patient before surgery, and both lower limbs were completely paralyzed including incontinence after surgery. Thoracic medulla MRI examination showed syringomyelia and long-level spinal cord ischemic changes. After comprehensive rehabilitation, only minimal lower limb sensation returned, and there was no recovery of motor function in the setting of complete spinal cord injury. Use of rehabilitation assistive devices improved the patients’ ability to perform activities of daily living. Scholars now generally believe that the spinal cord has plasticity like the brain.^[30]^ Research results have shown that functional training increases the expression of endogenous neurotrophic factors in patients with incomplete spinal cord injury, inducing the lateral branches of corticospinal tract budding, and promoting the recovery of motor function.^[31–34]^

It is imperative to be aware of these case limitations. Despite stressing the importance of early cerebrospinal fluid drainage, it was not performed in either case. Furthermore, lack of long-term follow-up and social reintegration guidance.

## 4. Conclusion

Perioperative spinal cord protection is directly related to postoperative quality of life. Once the symptoms of spinal cord ischemic injury occur, early detection and treatment should be done. Symptom stability flags referral to commence rehabilitation. Repetitive functional training is necessary to help patients return to the family and society as soon as possible.

## Acknowledgments

Thanks to all the reviewers and editors for their opinions and suggestions.

## Author contributions

**Writing – original draft:** Shu Cheng, Xuan Li.

**Writing – review & editing:** Yufei Zhang, Chenxi Liu, Yi Rao, Yang Zhang, Jinlun Wu, Jinwen Sun, E-Liisa Laakso.
